# Solidification of Municipal Solid Waste Incineration Fly Ash through Co-Mechanical Treatment with Circulation Fluidized Bed Combustion Fly Ash

**DOI:** 10.3390/ma13010141

**Published:** 2019-12-30

**Authors:** Zhengzhen Yao, Zhonghui Xu, Qin Shuai, Xiaoyue Chen, Zao Jiang, Xi Peng, Yu Li, Ran An, Xin Jiang, Han Li

**Affiliations:** Key Laboratory of Solid Waste Treatment and Resource Recycle, Ministry of Education, Southwest University of Science and Technology, Mianyang 621010, Sichuan, China; swustzhengzhenyao@163.com (Z.Y.); qinshuai_t@163.com (Q.S.); XiaoyueChenV@163.com (X.C.); 18281612117@163.com (Z.J.); pengxi2014@163.com (X.P.); ly1213424918@163.com (Y.L.); an15528535995@163.com (R.A.); jiangxin7777@foxmail.com (X.J.); lihan1099599@163.com (H.L.)

**Keywords:** MSWIFA, CFBCFA, solidification, co-mechanical treatment, heavy metals

## Abstract

This study aims to explore the solidification performance of municipal solid waste incineration fly ash (MSWIFA) through co-mechanical treatment with circulation fluidized bed combustion fly ash (CFBCFA). The mineral characterization, physical properties, and leaching resistance of the solidified bodies are investigated by X-ray diffraction spectroscopy (XRD), Fourier transform infrared spectroscopy (FT-IR), Thermogravimetry-differential thermal analysis (TG-DTA), compressive strength, porosity, and leaching test, respectively. C–S–H, ettringite (AFt), and Friedel’s salt (FS) are the predominant hydrate products in the CFBCFA based solidified bodies, which are similar to the cement based solidified bodies. However, CFBCFA based solidified bodies exhibit higher compressive strength (36.7 MPa) than cement based solidified bodies (11.28 MPa), attributing to the three reasons: lower porosity and more compact internal structure of CFBCFA based solidified bodies; large amounts of Ca(OH)_2_ originating from MSWIFA are conducive to promoting the hydration reaction extent and compressive strength of the CFBCFA based solidified bodies; excessive Ca(OH)_2_ would cause compressive strength deterioration for the cement based solidified bodies. The heavy metals (Zn, Cu, Cr, Cd, and Pb) concentrations in the extraction solution of the CFBCFA based solidified bodies are far below the requirements of Chinese National Standard GB 5085.3-2007. The solidification of MSWIFA through co-mechanical treatment could be an ideal substitute for cement solidification technology.

## 1. Introduction

MSWIFA generates from waste incinerators, which is regarded as hazardous solid waste due to the enrichment of easily leachable heavy metals and, in some cases, potentially organic pollutants [1]. To safely handle and dispose of MSWIFA, various treatment have been proposed including recovery of valuable component, stabilization with wood pellet ash, and solidification with cement [2,3]. Currently, cement solidification technology is widely proposed and investigated to deal with MSWIFA owing to its technical feasibility [4,5]. Unfortunately, cement production has defects of significant equipment investment, high energy consumption, high operating cost, and environmental pollution [6]. Therefore, more suitable material should be considered to replace cement to solidify MSWIFA.

The fly ash coming from circulation fluidized bed combustion boilers could encapsulate the heavy metals effectively due to the pozzolonicity and self-gelling properties and has the potential to be cement substitute to solidify MSWIFA [7,8]. Nevertheless, CFBCFA based solidified bodies would confront the risk of destructive expansion and strength reduction, which is related to the low soluble anhydrite and poor pozzolonicity of other components [9]. Thus, special measures should be taken into account to enhance the reactivity of CFBCFA.

Mechanical activation has been frequently applied in the field of preparing alkali-activated slag cement, which could increase the hydration reactivity of the solid waste by changing the particle size and specific surface [10,11,12,13]. As for CFBCFA, the grinding process could accelerate the hydration reaction of the anhydrite and other silica-alumina components, which is crucial to diminish the expansion of the hardened blocks [14,15]. Meanwhile, large amounts of calcium hydroxide and calcium oxide in MSWIFA not only provide an alkaline environment for the hydration reaction but also could be helpful to increase the pozzolonicity of the CFBCFA during co-mechanical treatment [16]. Moreover, co-mechanical treatment might reduce the porosity and improve the leaching resistance of the solidified bodies. Perhaps it is a practical method to solidify MSWIFA through co-mechanical treatment with CFBCFA.

As described above, the present study is undertaken to explore the solidification performance of MSWIFA through co-mechanical treatment with CFBCFA, and cement solidification technology is chosen for comparison. The mineralogical characterization, porosity, compressive strength, and leaching resistance of the solidified bodies are given full consideration.

## 2. Materials and Methods

### 2.1. Materials

**Figure 1:**
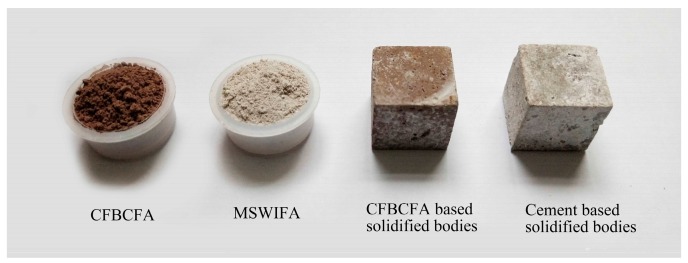
The appearance of MSWIFA, CFBCFA, CFBCFA and cement based solidified bodies.

**Figure 2:**
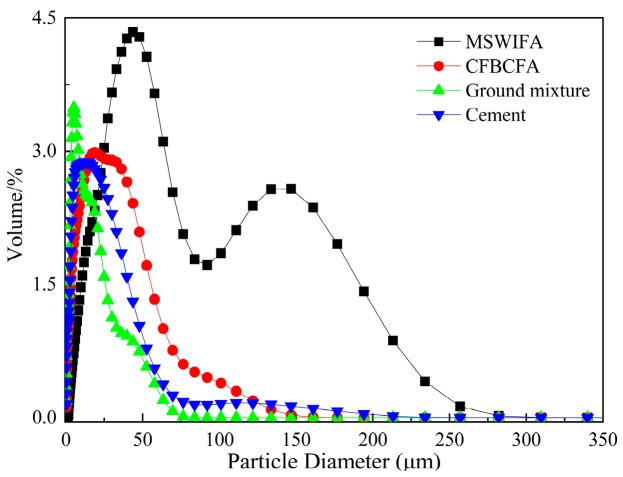
The particle size distributions of the raw materials and ground mixture.

The MSWIFA was supplied by Tongxing Waste Incineration Power Plant (Beibei District, Chongqing, China, Figure 1). The source of CFBCFA was Neijiang Baima Circulating Fluidized Bed Demonstration Power Station Co., Ltd. (Sichuan, China, Figure 1). The MSWIFA was mixed with CFBCFA at the weight ratio of 7:3. Then, the mixture was ground 5 h by high-energy ball milling (QM-WX4, Nanjing university instrument factory, 40 Hz). The Portland cement was produced by Zhonglian Cement Plant (Sichuan, China). The detailed chemical constituents of MSWIFA, CFBCFA, and Portland cement are present in Table 1. The particle size distributions (Figure 2) of the raw materials and ground mixture were measured by Beckman Coulter LS13320 laser diffraction analyzer. It was observed that the ground mixture showed a narrower particle size (d50 = 6.107 µm), which was much smaller than the MSWIFA, CFBCFA, and cement.

### 2.2. Sample Preparation

The CFBCFA based solidified bodies were prepared with ground mixture and water at the mass ratio of 1:0.3 (Table 2). Cement based solidified bodies were also used to prepare an additional solidification matrix according to a fixed mass ratio (Cement:MSWIFA:water = 0.3:0.7:0.3). The mix designs of the solidified bodies were shown in Table 2. The slurries were cast into 20 mm cube molds and vibration for 10 min to remove entrained air bubbles. Subsequently, the molds were sealed by polyethylene plastic films and kept for 24 h at a standard curing condition (20 ± 1 °C, ≥90% relative humidity). After 24 h, the samples were demolded and kept at the same conditions without the polyethylene film for a further 27 days. The prepared CFBCFA and cement based solidified bodies are shown in Figure 1.

### 2.3. Analytical Methods

#### 2.3.1. Mineralogical Characteristics

The mineralogical characterization of the solidified bodies was characterized via X-ray diffraction spectroscopy (XRD), Fourier transform infrared spectroscopy (FT-IR), and Thermogravimetry-differential thermal analysis (TG-DTA). The XRD pattern was obtained with an X’Pert PRO (PANalytical B.V., Almelo, The Netherlands) multifunctional X-ray diffractometer using CuKα radiation generated at 35 kV and 40 mA and a scanning rate of 10° per min from 3° to 80° (2θ). FT-IR spectrum in the 4000–400 cm^−1^ region was recorded by Nexus 670 infrared analyzer (PerkinElmer Scientific, Newark, New Jersey, USA) using the KBr pellet technique (0.5 mg powder sample mixed with 250 mg of KBr). The thermal behavior (TG-DTA) of the solidified bodies was tested by SDT Q160 synchronous thermal analyzer (TA Instruments, New Castle, DE, USA), which was heated from ambient to 1000 °C in a nitrogen atmosphere.

#### 2.3.2. Porosity and Compressive Strength

**Figure 3:**
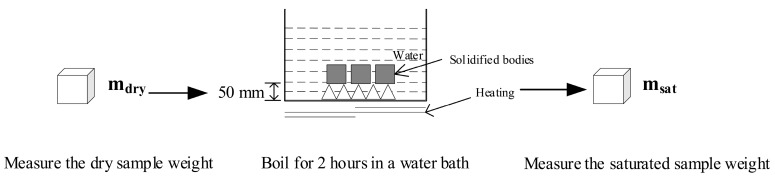
Schematic for measuring the porosity of CFBCFA and cement based solidified bodies.

The porosity of solidified bodies was determined by Archimedes’ principle [17] (Figure 3). Distilled water was used as the immersion medium during the procedure. After the curing period, the mass of the dried samples were determined (m_dry_); then, the samples were immersed into a Heat-gathering Style Magnetism Mixer (DF-101S) and boil for 5 h; after the sample was cooled for 14 h, the saturated mass of the samples after boiling was measured (m_sat_). From the knowledge of these masses, the effective porosity can be calculated following the standard of ASTM C642-2013. The compressive strength of solidified bodies was measured through a universal testing machine (CMT5504, Shanghai, China) according to the Chinese National Standard GB/T 17671-1999. The final results were obtained by calculating the average of six tests. Each test result was obtained by taking an average of the results from six specimens.

#### 2.3.3. Leaching

**Figure 4:**
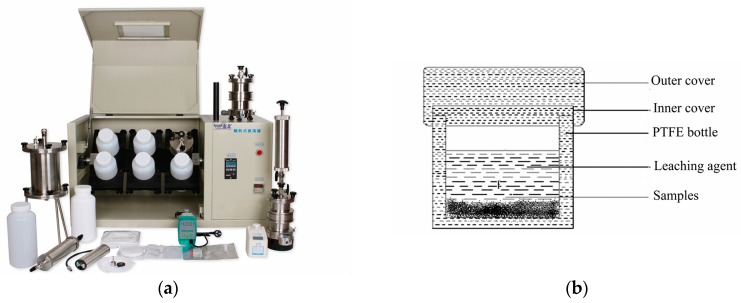
The device of leaching experimental, (**a**) Rotary Agitator; (**b**) extraction bottle.

The heavy metal leaching from solidified bodies and MSWIFA was performed in accordance with the Chinese standard HJ/T 300-2007. Typically, the crushed samples collected after the compressive strength test should be dried in an oven with 105 °C and ground in a ceramic mortar till the particle size was below 9.5 mm. Subsequently, the powder and leaching agent with a liquid/solid ratio of 20 was put into a 2000 mL extraction bottle, which was fixed on a tumble-type oscillating device for 18 h at 30 rpm (Figure 4). Finally, the extraction leachate was filtered through a vacuum filter and collected to measure the heavy metal contents by Atomic Absorption Spectroscopy (Analytik Jena, Jena, Germany).

## 3. Results and Discussion

### 3.1. Mineral Characterization

#### 3.1.1. X-Ray Diffraction Analysis

**Figure 5:**
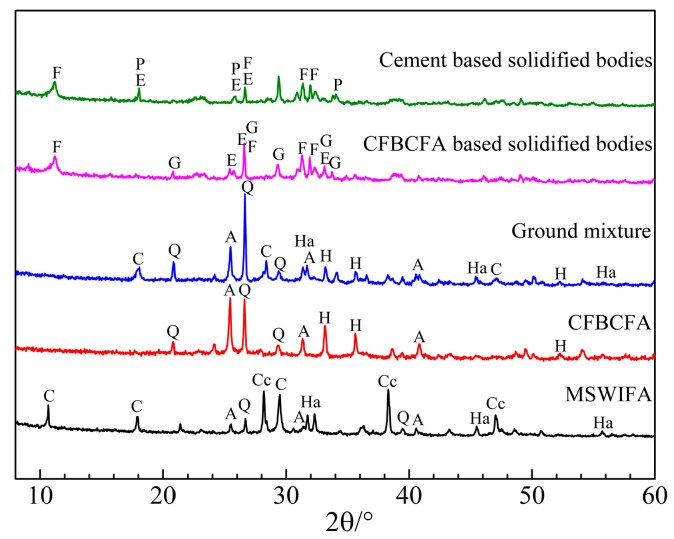
XRD patterns for the raw materials, ground mixture, and solidified bodies (A-Anhydrite; C-Calcite; E-Ettringite; F-Friedel’s salt; G-Gismondine; H-Hematite; Ha-Halite; P-Portlandite; Q-Quartz; Cc-Calcium hydroxyl chloride).

The XRD patterns of the raw materials, ground mixture, and solidified bodies are shown in Figure 5. The main crystalline compounds in MSWIFA are calcium hydroxyl chloride, calcite, halite, anhydrite, and quartz, whereas the principal mineral phases of CFBCFA are anhydrite, hematite, and quartz. Compared to CFBCFA, the peak intensity of anhydrite in the ground mixture is obviously weakened, indicating the serious crystalline phase destruction during the co-mechanical treatment [15]. In addition, Friedel’s salt (FS, Ca_3_Al_2_O_6_·CaCl_2_·10H_2_O) is detected in the CFBCFA based solidified bodies, which is ascribed to the hydration reaction of Al_2_O_3_ with chloride and Ca(OH)_2_ [18,19]. Meanwhile, ettringite (AFt) and gismondine are also found in the CFBCFA based solidified bodies, resulting from the hydration reaction between the Ca(OH)_2_ and the other active components in the ground mixture, which are helpful to hold the heavy metals in the crystalline structure with their ions exchange abilities [20,21]. Furthermore, C–S–H could be found in the CFBCFA and cement based solidified bodies, which is responsible for the strength development and heavy metals encapsulation [22]. In fact, FS, AFt and C–S–H are the predominant phases or hydration products in cement and CFBCFA based solidified bodies, implying that the similar hydration behavior occurs during the two solidification processes.

#### 3.1.2. Fourier Transform Infrared Analysis

**Figure 6:**
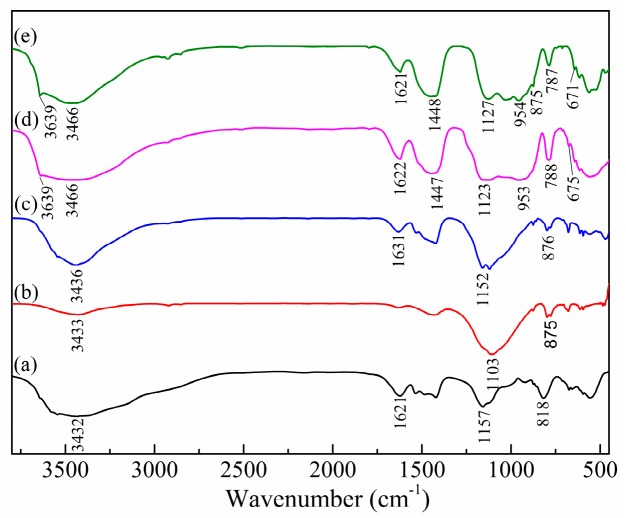
FT-IR spectrums for the raw materials (a-MSWIFA, b-CFBCFA), ground mixture (c), and solidified bodies (d-CFBCFA based solidified bodies, e-Cement based solidified bodies).

As can be observed in Figure 6, two broad bands around 3433 and 1631 cm^−1^ in all FT-IR spectrums are corresponded to the O–H stretching vibration of hydration water and H–O–H bending vibration of interlayer water, respectively [23]. In Figure 6d,e, the band around 3639 cm^−1^ (O–H bending vibration) is associated with the portlandite [24]. The band around 953 cm^−1^ is attributed to the Si–O–Si asymmetric stretching vibration, which could be correlated to C–S–H or gismondine [25,26]. The adsorption bands around 1123 cm^−1^ (S–O asymmetric vibration) together with 675 cm^−1^ (Al–OH bending vibration) represent the presence of AFt [5]. The Al-OH bending vibration is detected around 788 cm^−1^ and always considered to be associated with FS [5,27]. The band around 875 and 1448 cm^−1^ are identified as the bending vibration and stretching vibration of CO_3_^2−^ [24]. FTIR analysis further demonstrates that C–S–H, AFt and Fs are the dominant hydration products in cement and CFBCFA based solidified bodies, which is consistent with the XRD result.

#### 3.1.3. Thermogravimetry-Differential Thermal Analysis

**Figure 7:**
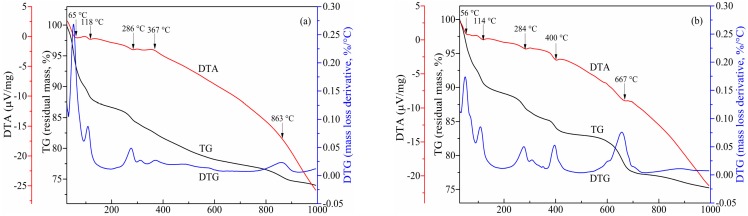
TG-DTA curves for solidified bodies (**a**)-CFBCFA based solidified bodies; (**b**)-cement based solidified bodies.

From the curves in Figure 7, there is a continuous weight decline of the both solidified bodies with about 25% of total weight loss over the whole testing temperature range. The endothermic peaks occurred before 200 °C with around 13% weight loss are associated with the free water evaporation and the dehydration of C–S–H, AFt and FS [28,29,30]. The less pronounced shoulder around 286 °C can be interpreted by the dehydration of small quantities of hydrocalumite [30,31]. At around 367 °C in Figure 4a, a very small endothermic peak and weight loss correlated to the dehydration of portlandite are detected, yet the peak in Figure 4b exhibits even stronger and shift to 400 °C since a relatively large amount of portlandite in cement based solidified bodies [32,33]. A large proportion of Ca(OH)_2_ originating from MSWIFA may not fully attend the main hydration reaction and is harmful to the compressive strength development of the cement based solidified bodies. The inconspicuous endothermic peak near 863 °C accompanied by weight loss (approximately 7%) in Figure 4a can be ascribed to the decomposition of CaCO_3_, while the peak appears at 667 °C in Figure 4b due to the more quantities of CaCO_3_ in cement based solidified bodies due to the large quantities of Ca(OH)_2_ in cement based solidified bodies [34]. The excessive Ca(OH)_2_ in cement based solidified bodies indicates that large amounts of calcium hydroxide and calcium oxide haven’t joined in the dominant hydration reactions during the cement solidification process, which is harmful to the strength development of the solidified bodies.

### 3.2. Porosity and Compressive Strength

The compressive strength of CFBCFA based solidified bodies (36.70 MPa) is much higher than that of cement based solidified bodies (11.28 MPa), which is correlated to the lower porosity (Table 3) and more compact internal structure of CFBCFA based solidified bodies. As shown in Table 3, the porosity of CFBCFA and cement based solidified bodies are 18% and 24.5%, respectively, which could be related to the w/c ratio and degree of hydration reaction for the matrices [35]. In practice, a significant decrease of the particle size of the ground mixture (Figure 2) is conducive to forming an even denser structure for CFBCFA based solidified bodies. Meanwhile, co-mechanical treatment increases the pozzolonicity and enhances the hydration reaction extent, which is beneficial to the strength development of CFBCFA based solidified bodies [36]. Moreover, huge amounts of Ca(OH)_2_ originating from the MSWIFA do not completely participate in the formation of C–S–H during the hydration process, which would cause compressive strength deterioration for the cement based solidified bodies. Compared with other studies [37,38,39], solidification of MSWIFA through co-mechanical treatment with CFBCFA also exhibits excellent mechanical performance, and seems to more suitable as a substitute for cement solidification technology.

### 3.3. Leaching

In Table 4, the leaching concentrations of heavy metals in MSWIFA are sharply declining and far below the permitted values of Chinese National Standards GB 5085.3-2007 after solidification treatment, indicating the remarkable leaching resistance of CFBCFA and cement based solidified bodies. Hence, solidification of MSWIFA through co-mechanical treatment with CFBCFA could be an ideal substitute for cement solidification technology. Actually, the solidification mechanisms of the heavy metals in CFBCFA based solidified bodies could be divided into three processes: physical encapsulation, chemical absorption, and precipitation. Firstly, C–S–H forms a barrier on the surface of MSWIFA particles during the hydration process, restricting the migration of heavy metal ions through mechanical processes or chemical reactions [40]. Secondly, ettringite and gismondine in solidified bodies may adsorb heavy metals through ions exchange [41,42]. Thirdly, a high alkalinity environment during the solidification process probably helps to lock heavy metals in the shape of precipitation [43].

## 4. Conclusions

This work investigates the solidification performance of MSWI fly ash through co-mechanical treatment with CFBC fly ash, compared to the cement solidification technology. The results prove that the solidification of MSWIFA through co-mechanical treatment could be an ideal substitute for cement solidification technology. The main conclusions could be drawn as follows:

1. The CFBCFA based solidified bodies exhibit better compressive strength than cement based solidified bodies. The compressive strength of CFBCFA based solidified bodies measures up to 36.7 MPa after 28 d curing, while cement based solidified bodies could only reach 11.28 MPa, which is correlated to the lower porosity and more compact internal structure of CFBCFA based solidified bodies;
2. XRD, FT-IR, and TG-DTA analyses indicate that the predominant hydrate products in CFBCFA and cement based solidified bodies are C–S–H, AFt, and FS. Moreover, large amounts of Ca(OH)_2_ originating from MSWIFA are conducive to promoting the hydration reaction extent and compressive strength of the CFBCFA based solidified bodies, while excessive Ca(OH)_2_ is harmful to the compressive strength development of the cement based solidified bodies;
3. CFBCFA based solidified bodies possess excellent leaching resistance. The heavy metals (Zn, Cu, Cr, Cd, and Pb) concentrations in the extraction solution are far below the requirements of Chinese National Standard GB 5085.3-2007. The heavy metals in CFBCFA based solidified bodies are immobilized mainly depending on a combination of physical encapsulation and chemical absorption. In addition, partial heavy metal ions are locked in the form of precipitation due to the high alkalinity of the reaction system.

## Figures and Tables

**Table 1:** Chemical constituents of MSWIFA, CFBCFA, and Portland cement (wt%).

Component	CaO	Cl	Na_2_O	SO_3_	K_2_O	SiO_2_	Fe_2_O_3_	Al_2_O_3_	MgO	Others
MSWIFA	48.22	19.70	9.87	7.02	5.16	3.24	1.44	-	-	5.17
CFBCFA	9.64	-	0.44	7.96	-	45.60	15.16	17.10	0.98	3.12
Portland Cement	49.82	-	0.59	2.78	1.28	29.89	4.04	8.15	2.20	1.25

**Table 2:** The mix designs of CFBCFA/Cement based solidified bodies (wt%).

Material	Ground Mixture	Cement	MSWIFA	Water
CFBCFA based solidified bodies	100	-	-	30
Cement based solidified bodies	-	30	70	30

**Table 3:** The porosity and compressive strength of solidified bodies.

Material	Compressive Strength (MPa)	SD-c ^1^ (MPa)	Porosity (%)	SD-p ^2^ (%)
CFBCFA based solidified bodies	36.70	1.09	18.00	0.45
Cement based solidified bodies	11.28	0.55	24.50	0.19

^1^ SD-c: standard deviation of compressive strength, ^2^ SD-p: standard deviation of porosity.

**Table 4:** Leaching results for MSWIFA and solidified bodies (mg/L).

Elements	GB 5085.3-2007	MSWIFA	CFBCFA Based Solidified Bodies	Cement Based Solidified Bodies
Zn	100.00	85.8300	0.5086	0.0358
Pb	5.00	2.9457	0.0519	0.0939
Cu	100.00	18.0450	0.6462	0.1097
Cd	1.00	5.1914	0.1222	0.0998
Cr	5.00	16.0800	0.6145	0.8734
